# Supplementary figures and images for: Zika virus infection differentially affects genome-wide transcription in neuronal cells and myeloid dendritic cells
Source: PLoS One. 2020 Apr 14;15(4):e0231049. doi: 10.1371/journal.pone.0231049 (PMC7156053; doi:10.1371/journal.pone.0231049)

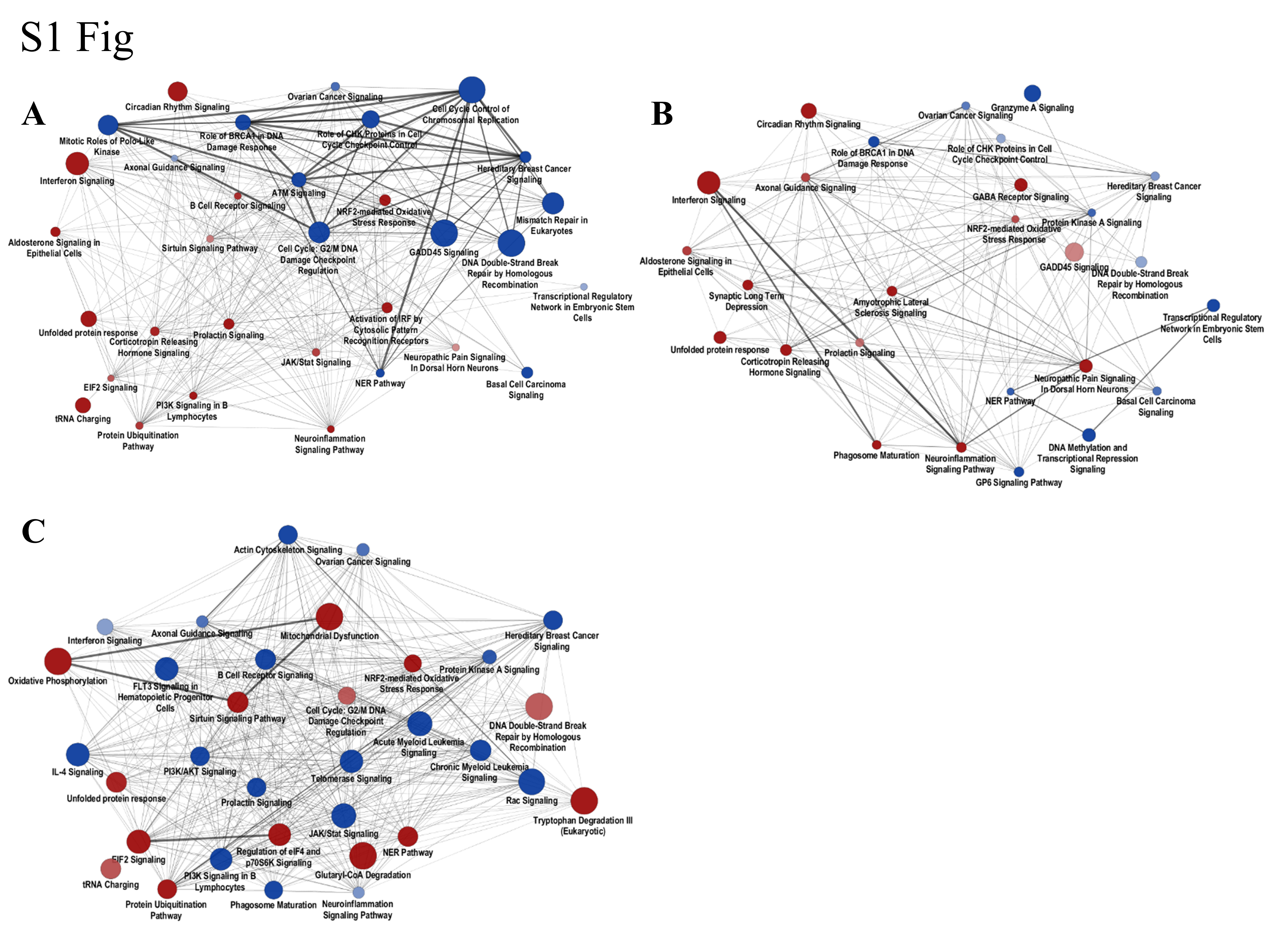

Supplement: S1 Fig — Protein-protein interactions were grouped by significantly expressed biological pathways in hPNs (A), hNCCs (B), and mDCs (C). The nodes represent biological pathways, and node size depends on the betweenness centrality score calculated by network analysis. Edges were generated based on interactions of DEGs contained in pathways. Red: upregulated pathways; blue: downregulated pathways. The networks were generated with v3.7.0 Cytoscape. (TIF) [file pone.0231049.s004.tif]

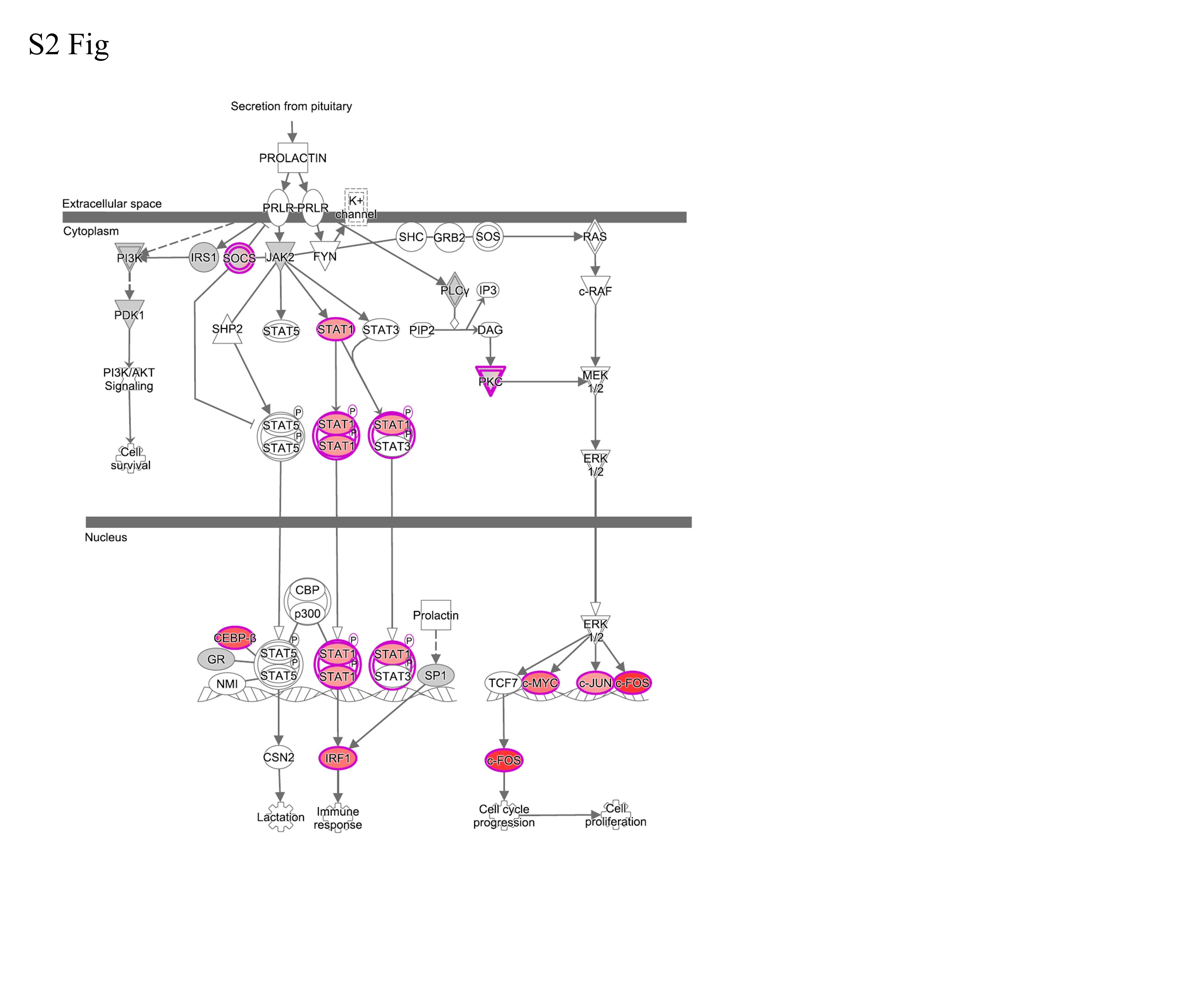

Supplement: S2 Fig — Prolactin signaling consists of JAK/Stat signaling, PI3K signaling, and RAS cascade. (TIF) [file pone.0231049.s005.tif]
