# Supplementary material for: Zika virus infection differentially affects genome-wide transcription in neuronal cells and myeloid dendritic cells
Source: PLoS One. 2020 Apr 14;15(4):e0231049. doi: 10.1371/journal.pone.0231049 (PMC7156053; doi:10.1371/journal.pone.0231049)
Supplement: Attachment — Submitted filename: rebuttal letter.docx [file pone.0231049.s006.docx]

PONE-D-19-21064
Zika virus infection differentially affects genome-wide transcription in neuronal cells and macrophages
PLOS ONE

Dear Dr. Park,

Thank you for submitting your manuscript to PLOS ONE. After careful consideration, we have decided that your manuscript does not meet our criteria for publication and must therefore be rejected.

I agree with reviewers that the difference in cell type and virus strain could be crucial. Other concerns raised are also significant and some might not be remedied.

I am sorry that we cannot be more positive on this occasion, but hope that you appreciate the reasons for this decision.

Yours sincerely,

Dong-Yan Jin
Academic Editor
PLOS ONE

Additional Editor Comments:
I have no other option in light of the criticisms made by reviewers.


[Note: HTML markup is below. Please do not edit.]

Reviewers' comments:

Reviewer's Responses to Questions

**Comments to the Author**

1. Is the manuscript technically sound, and do the data support the conclusions?

The manuscript must describe a technically sound piece of scientific research with data that supports the conclusions. Experiments must have been conducted rigorously, with appropriate controls, replication, and sample sizes. The conclusions must be drawn appropriately based on the data presented.

Reviewer #1: Yes

Reviewer #2: Partly

2. Has the statistical analysis been performed appropriately and rigorously?

Reviewer #1: Yes

Reviewer #2: I Don't Know

3. Have the authors made all data underlying the findings in their manuscript fully available?

The [PLOS Data policy](http://www.plosone.org/static/policies.action#sharing) requires authors to make all data underlying the findings described in their manuscript fully available without restriction, with rare exception (please refer to the Data Availability Statement in the manuscript PDF file). The data should be provided as part of the manuscript or its supporting information, or deposited to a public repository. For example, in addition to summary statistics, the data points behind means, medians and variance measures should be available. If there are restrictions on publicly sharing data—e.g. participant privacy or use of data from a third party—those must be specified.

Reviewer #1: Yes

Reviewer #2: Yes

4. Is the manuscript presented in an intelligible fashion and written in standard English?

PLOS ONE does not copyedit accepted manuscripts, so the language in submitted articles must be clear, correct, and unambiguous. Any typographical or grammatical errors should be corrected at revision, so please note any specific errors here.

Reviewer #1: Yes

Reviewer #2: Yes

5. Review Comments to the Author

Please use the space provided to explain your answers to the questions above. You may also include additional comments for the author, including concerns about dual publication, research ethics, or publication ethics. (Please upload your review as an attachment if it exceeds 20,000 characters)

Reviewer #1: The manuscript entitled “Zika virus infection differentially affects genome-wide transcription in neuronal cells and macrophages” by Park et. al. describes how ZIKA virus infection affects the transcriptome of neurons, neural crest cells, neural stem cells and macrophages by analyzing published RNA seq data. In this study authors did comparative network analysis by protein-protein interaction predication. Authors found that major gene set are affected by ZIKV infection is related to neuroinflammation, interferon signaling, cell cycle, and DNA repair pathways. But this type of study already known and published showing involvement of these pathways in dysregulation of neural stem cell survival and neuronal cell death. I could not find the novel gene or proteins which affects the neuronal survival after ZIKV infection, which could target later to further validate their involvement. Overall the manuscript is nicely compiled and tried to find out factors that affecting the neural stem cell population during ZIKV infection, but there are many serious concerns raised in the manuscript which need to address to strengthen the manuscript:

1. Authors should need to find out the gene set and pathways which are not earlier published.

**🡪 Exactly, we found out novel pathways and focused genes by ZIKV infection. Especially, prolactin signaling pathway including JAK/STAT, PI3K, RAS pathways were downregulated myeloid dendritic cell by ZIKV infection. Also, the pathways were revealed by the comparison analysis between neuronal cells and mDCs, because the gene showed the highly different gene expression pattern. For example, *IRS2* (hPNs: 1.31, hNCCs: 1.16, mDCs: −0.35 log_2_[fold-change]), *PIK3C3* (hPNs: 0.09, hNCCs: 0.45, mDCs: −1.20), *PDPK1* (hPNs: 0.43, hNCCs: 0.16, mDCs: −2.08), *STAT3* (hPNs: 0.41, hNCCs: 0.43, mDCs: −1.72), and *IRF1* (hPNs: 2.56, hNCCs: 1.77, mDCs: −1.41), etc. The more genes were also described on the result in manuscript.**

**Interestingly, DNA repair system including the NER pathway were only upregulated in mDC with *TOP2B*, *USP7*, *CETN2*, *RPA2*, and *COPS2*. Also, DNA Double-Strand Break Repair was upregulated in mDC, but not in neuronal cells.**

**Additionally, oxidative phosphorylation and mitochondrial dysfunction share many genes which were significantly upregulated in mDCs, but not in neurons. Details of the genes are as follows. Oxidative phosphorylation, known as cellular respiration, occurs in the mitochondria, where enzymes catalyze the generation of ATP and the transfer of electrons to molecular oxygen. The upregulation of oxidative phosphorylation can lead to escape of the immune defense in mDCs from ZIKV infection.**

2. In figure 1C, groups in vein diagram could not read.

**🡪 The figure 1C was changed more clearly.**

3. All the figures in the manuscript are not very clear to read and understand what does it mean. Specially figure 3 and Fig 4 are not conclusive. Authors should need to take care of image pixel and size, so readers could understand the contents.

**🡪 Sorry about that. We tried to improve the image pixel and size for figure 3 and 4.**

4. Does authors find the genes involved in ER stress due to ZIKV infection in different cell types?

**🡪 In previous our research on comparison between neuronal cells and macrophages, ER stress-related genes were dysregulated in only macrophage by ZIKV infection. From the results, we suggested that ZIKV could lead the activation of phagocytosis including phagosome, endoplasmic reticulum, and clathrin-mediate vehicle in neuronal cells.**

**However, the characteristic did not appear in the re-analysis with neuronal cells and myeloid dendritic cells. The re-analysis showed that the upregulated genes only in neuronal cells were abundant in clathrin-sculpted gamma-aminobutyric acid transport vesicle membrane (GO:0061202), clathrin-sculpted gamma-aminobutyric acid transport vesicle (GO:0061200), and endocytic vesicle lumen (GO:0071682).**

**By previous researches, ZIKV is introduced into host cells using clathrin-mediated endocytic mechanisms after binding to the cell surface receptors, and is transported to endosomes [1]. The viral RNA, after entry into the cell is translated to a larger polyprotein on the endoplasmic reticulum. After a few steps, the newly synthesized virus traffic from the endoplasmic reticulum to the surface through the Golgi to spread the infection to nearby cells [2]. Our result supported that ZIKV could lead the activation of phagocytosis including phagosome,**

5. Authors could also perform the (Gene set enrichment analysis) GSEA analysis in each groups.

**🡪 Yes, we performed GSEA analysis in each group. The data is available S3 table.**

Reviewer #2: In this manuscript, Park et al reported the differential effect of ZIKV infection in genome-wide transcription between neuronal cells and macrophage. By comparison of RNA-seq data acquired from ZIKV-infected neuronal cells and macrophage, they found several differentially expressed genes from various signaling pathways, such as interferon and cell cycle progression, between two groups of cells. These findings further enriched current knowledge about the pathogenesis of neuronal symptom caused by ZIKV infection.

A major problem in this research might highly weaken its significance in the field. Specifically, the RNA-seq data used were obtained from two groups of cells, neuronal cell and macrophage. However, these cells were infected with different strains of ZIKV --the neuronal cells were infected with strains isolated from Puerto Rico or Mexico in 2015 while macrophage was infected with virus isolated from Cambodia in 2010. We know that ZIKV isolated from Cambodia in 2010 was with weak neurovirulence comparing with 2015 strains (https://science.sciencemag.org/content/358/6365/933). To this end, the comparison of gene expression change between the two groups of cells is clearly less meaningful. It is possible that this differential change in gene transcription is caused by the different virulence of ZIKV strains but not due to the cell type issues. If authors tried to compare the differential change in gene transcription induced by ZIKV among different cell types, it is absolutely better to infect cells with same strain, or at least the evolutionary correlation and virulence between strains should be close to each other. Also, results acquired from network analysis need to be validated.

**🡪 As your comments, we re-analyzed the data set using same strain of ZIKV (PRVABC59) which isolated from Puerto Rico in 2015. The PRVABC59 strain was reported as serious neuronal damage [3]. After then, we re-analyzed each conditions of peripheral neurons, neural crest cells, and myeloid dendritic cell.**

**The comparative network analysis can give us novel gene such as NBN, RPA2, CSF1R, and SOCS5. The several genes including TOP2B, OAS1, IRF1, STAT3, IRS2, SOCS1, and RELA were presented for important target in both neuronal cells and mDCs after ZIKV infection. The expression changes of genes consisting network had the statistical validation by p-value and false discovery rate.**

6. PLOS authors have the option to publish the peer review history of their article ([what does this mean?](https://journals.plos.org/plosone/s/editorial-and-peer-review-process#loc-peer-review-history)). If published, this will include your full peer review and any attached files.


If you choose “no”, your identity will remain anonymous but your review may still be made public.


**Do you want your identity to be public for this peer review?** For information about this choice, including consent withdrawal, please see our [Privacy Policy](https://www.plos.org/privacy-policy).

Reviewer #1: Yes: Shashi Kant Tiwari, Ph.D., University of California, San Diego, USA

Reviewer #2: No


[NOTE: If reviewer comments were submitted as an attachment file, they will be attached to this email and accessible via the submission site. Please log into your account, locate the manuscript record, and check for the action link "View Attachments". If this link does not appear, there are no attachment files to be viewed.]

- - - - -
For journal use only: PONEDEC3

**Reference**

1.	Persaud M, Martinez-Lopez A, Buffone C, Porcelli SA, Diaz-Griffero F. Infection by Zika viruses requires the transmembrane protein AXL, endocytosis and low pH. Virology. Academic Press; 2018;518: 301–312. doi:10.1016/J.VIROL.2018.03.009

2.	Savidis G, McDougall WM, Meraner P, Perreira JM, Portmann JM, Trincucci G, et al. Identification of Zika Virus and Dengue Virus Dependency Factors using Functional Genomics. Cell Rep. Cell Press; 2016;16: 232–246. doi:10.1016/J.CELREP.2016.06.028

3.	Kawai Y, Nakayama E, Takahashi K, Taniguchi S, Shibasaki KI, Kato F, et al. Increased growth ability and pathogenicity of American- and Pacific-subtype Zika virus (ZIKV) strains compared with a Southeast Asian-subtype ZIKV strain. PLoS Negl Trop Dis. NLM (Medline); 2019;13: e0007387. doi:10.1371/journal.pntd.0007387
