# Supplementary material for: Zika virus infection differentially affects genome-wide transcription in neuronal cells and myeloid dendritic cells
Source: PLoS One. 2020 Apr 14;15(4):e0231049. doi: 10.1371/journal.pone.0231049 (PMC7156053; doi:10.1371/journal.pone.0231049)
Supplement: Attachment — Submitted filename: rebuttal letter_v2.docx [file pone.0231049.s007.docx]

Dear Prof. Dong-Yan Jin.

Here is the revision about "PONE-D-19-31016 Zika virus infection differentially affects genome-wide transcription in neuronal cells and myeloid dendritic cells". Our paper is valuable to understand the interaction of host cells and viruses such as ZIKV. In the paper, we focused on the significant difference gene sets when ZIKV enter into neuronal cells and immune cell using comparative network analysis.

The methodology and systemic approaches will be helpful to prevent emerging viruses such as SARS-CoV-2. In addition, RNAseq has a high correlation experimental qPCR with over 85%. We believe the result of ZIKV infection between neuronal cells and the immune cell can give new clues for developing ZIKV drugs and vaccines.

Sincerely yours,

Daeui Park

PONE-D-19-31016

Zika virus infection differentially affects genome-wide transcription in neuronal cells and myeloid dendritic cells

PLOS ONE

Reviewers' comments:

Reviewer's Responses to Questions

Comments to the Author

1. Is the manuscript technically sound, and do the data support the conclusions?

The manuscript must describe a technically sound piece of scientific research with data that supports the conclusions. Experiments must have been conducted rigorously, with appropriate controls, replication, and sample sizes. The conclusions must be drawn appropriately based on the data presented.

Reviewer #1: Partly

Reviewer #2: Yes

2. Has the statistical analysis been performed appropriately and rigorously?

Reviewer #1: Yes

Reviewer #2: Yes

3. Have the authors made all data underlying the findings in their manuscript fully available?

The PLOS Data policy requires authors to make all data underlying the findings described in their manuscript fully available without restriction, with rare exception (please refer to the Data Availability Statement in the manuscript PDF file). The data should be provided as part of the manuscript or its supporting information, or deposited to a public repository. For example, in addition to summary statistics, the data points behind means, medians and variance measures should be available. If there are restrictions on publicly sharing data—e.g. participant privacy or use of data from a third party—those must be specified.

Reviewer #1: Yes

Reviewer #2: Yes

4. Is the manuscript presented in an intelligible fashion and written in standard English?

PLOS ONE does not copyedit accepted manuscripts, so the language in submitted articles must be clear, correct, and unambiguous. Any typographical or grammatical errors should be corrected at revision, so please note any specific errors here.

Reviewer #1: Yes

Reviewer #2: Yes

5. Review Comments to the Author

Please use the space provided to explain your answers to the questions above. You may also include additional comments for the author, including concerns about dual publication, research ethics, or publication ethics. (Please upload your review as an attachment if it exceeds 20,000 characters)

Reviewer #1: The manuscript entitled “Zika virus infection differentially affects genome-wide transcription in neuronal cells and myeloid dendritic cells” by Park et. al. analyzed RNA sequencing data from ZIKV-infected neuronal cells and myeloid dendritic cells by comparative network analyses using protein-protein interaction information, and revealed that some major genes related to DNA repair systems, prolactin signaling as well as the interferon signaling, neuroinflammation, and cell cycle pathways showed differential changes in the peripheral neurons, neural crest cells, and myeloid dendritic cells. These findings further enriched current knowledge about the pathogenesis of ZIKV infection, but some serious concerns still need to be addressed before publication.

1. Some gene set and pathways in this manuscript were earlier published, so the authors should point out and focus on the novel genes and pathways they found using comparative network analysis.

Our paper represented novel pathways as well as already published pathways in ZIKV infection. Major pathways are related to neuroinflammation, interferon signaling, and cell cycle pathways. In addition, we found out novel pathways and focused genes by ZIKV infection.

Especially, prolactin signaling pathway including JAK/STAT, PI3K, RAS pathways were downregulated myeloid dendritic cell by ZIKV infection. The pathways were revealed by the comparison analysis between neuronal cells and mDCs, because the gene showed the highly different gene expression pattern. For example, *IRS2* (hPNs: 1.31, hNCCs: 1.16, mDCs: −0.35 log_2_[fold-change]), *PIK3C3* (hPNs: 0.09, hNCCs: 0.45, mDCs: −1.20), *PDPK1* (hPNs: 0.43, hNCCs: 0.16, mDCs: −2.08), *STAT3* (hPNs: 0.41, hNCCs: 0.43, mDCs: −1.72), and *IRF1* (hPNs: 2.56, hNCCs: 1.77, mDCs: −1.41), etc. The more genes were also described on the result in manuscript.

Interestingly, DNA repair system including the NER pathway were only upregulated in mDC with *TOP2B*, *USP7*, *CETN2*, *RPA2*, and *COPS2*. Also, DNA Double-Strand Break Repair was upregulated in mDC, but not in neuronal cells.

Additionally, oxidative phosphorylation and mitochondrial dysfunction share many genes which were significantly upregulated in mDCs, but not in neurons. Details of the genes are as follows. Oxidative phosphorylation, known as cellular respiration, occurs in the mitochondria, where enzymes catalyze the generation of ATP and the transfer of electrons to molecular oxygen. The upregulation of oxidative phosphorylation can lead to escape of the immune defense in mDCs from ZIKV infection.

2. All results were acquired from network analysis, it's better to choose some top hit new genes and further validate them by experiments.

Network analysis can give us the benefit to understand the mechanism about interaction between viruses and host cells. Therefore, many approaches have applied network analysis as well as top hit method. Our paper was based on RNAseq data of same strain of ZIKV (PRVABC59) which isolated from Puerto Rico in 2015. Because the RNAseq methods have high gene expression correlations with qPCR data with over 85% [1], the genes could show consistent results between RNA-sequencing and qPCR.

Reviewer #2: The manuscript by Park et. al. entitled “Zika virus infection differentially affects genome-wide transcription in neuronal cells and myeloid dendritic cells” describes how ZIKA virus infection affects the transcriptome of human pluripotent stem cells derived peripheral neurons, neural crest cells and myeloid dendritic cells by analyzing published RNA seq data. In this study authors did comparative network analysis by protein-protein interaction predication. Authors found that major gene set are affected by ZIKV infection is DNA repair systems and prolactin signaling as well as the interferon signaling, neuroinflammation, and cell cycle pathways. By reanalysis of data from previous submission, authors find some interesting immune and mitochondrial dysfunction genes in myeloid dendritic cells. Overall the manuscript is well written and authors tried to find out the novel pathways such as prolactin signaling and mechanisms of Zika virus infection in these cell types. But authors should need to take care of following points:

1. The figures 3, 4 and 5 in the manuscript are completely unable to understand and read properly to reach any conclusion.

As your comments, we changed figure 3 to clearly understand major gene set between neural cells and myeloid dendritic cells. Also, major gene were highlighted in figure 4 and 5.

2. In fig-1A, its looks like human pluripotent stem cells infected by ZIKV and then differentiated into neuronal cell types. But it should be like hPSC differentiated hPN and hNCC infected by ZIKV.

Correct, we changed the figure 1A. Thank you for your points.

3. Authors should need to discuss in details about the genes involved in ZIKA infection mediated mitochondria dysfunction, oxidative phosphorylation and prolactin signaling genes (IRS2, PIK3C3, JAK3, STAT3, and IRF1) as mentioned in results sections.

More detail the information of gene was added in results section. Already, interferon signaling is known to major gene set in ZIKV infection. However, prolactin signaling could be regarded as novel candidates by ZIKV infection. Prolactin signaling is related to stimulate the secretion of cytokines and expression of cytokine receptors in the immune system which is consisted of PI3K signaling and JAK/Stat signaling. Interestingly, the IRS2, PIK3C3, JAK3, STAT3, and IRF1 which belong to prolactin signaling was significantly downregulated only in mDCs, not neural cell. In addition, STAT3 may be a candidate for immune suppression by ZIKV infection, as several viruses reduced cellular STAT3 by promoting proteasomal degradation, and NS1 protein of dengue-2 virus directly interacted with human STAT3 protein. We discussed the points in result and discussion of the manuscript.

**Reference**

1. Everaert C et al. Benchmarking of RNA-sequencing analysis workflows using whole-transcriptome RT-qPCR expression data. Sci Rep. 2017 May 8;7(1):1559
